# Characteristics of Inorganic–Organic Hybrid Membranes Containing Carbon Nanotubes with Increased Iron-Encapsulated Content for CO_2_ Separation

**DOI:** 10.3390/membranes12020132

**Published:** 2022-01-21

**Authors:** Aleksandra Rybak, Aurelia Rybak, Waldemar Kaszuwara, Sławomir Boncel, Anna Kolanowska, Spas D. Kolev

**Affiliations:** 1 Faculty of Chemistry, Silesian University of Technology, Strzody 7, 44-100 Gliwice, Poland; slawomi.boncel@polsl.pl (S.B.); anna.kolanwska@polsl.pl (A.K.); 2 Faculty of Mining, Safety Engineering and Industrial Automation, Silesian University of Technology, 44-100 Gliwice, Poland; aurelia.rybak@polsl.pl; 3 Faculty of Materials Science and Engineering, Warsaw University of Technology, 00-661 Warszawa, Poland; waldemar.kaszuwara@pw.edu.pl; 4 School of Chemistry, The University of Melbourne, Parkville, VIC 3010, Australia; s.kolev@unimelb.edu.au

**Keywords:** CO_2_ separation, inorganic–organic hybrid membranes, mechanical properties, magnetic measurements, thermal analysis

## Abstract

Novel inorganic–organic hybrid membranes Fe@MWCNT/PPO or Fe@MWCNT-OH/SPPO (with a new type of CNTs characterized by increased iron content 5.80 wt%) were synthesized for CO_2_ separation. The introduction of nanofillers into the polymer matrix has significantly improved the hybrid membrane’s gas transport (D, P, S, and **α_*CO*_2_/*N*_2__**), magnetic, thermal, and mechanical parameters. It was found that magnetic casting has improved the alignment and dispersion of Fe@MWCNTs. At the same time, CNTs and polymer chemical modification enhanced interphase compatibility and the membrane’s CO_2_ separation efficiency. The thermo-oxidative stability and mechanical and magnetic parameters of composites were improved by increasing new CNTs loading. Cherazi’s model turned out to be suitable for describing the CO_2_ transport through analyzed hybrid membranes.

## 1. Introduction

Nowadays, the main problems with climate changes and global warming are caused due to a significant increase in greenhouse gases concentration in the atmosphere, mainly CO_2_ [1]. In the last century, the rising CO_2_ concentration was caused by increasing activity within energy production from fossil fuels, transport [2,3], and production of raw materials (steel, cement, etc.) [1].

The primary strategy for reducing CO_2_ emissions is Carbon Capture and Sequestration (CCS), including post-combustion, oxyfuel, pre-combustion processes, and direct air carbon capture [2,4]. CO_2_ separation is also significant in other applications, such as biogas production and natural gas sweetening [3,5,6,7,8]. Such separated CO_2_ can then be used in enhanced oil recovery (EOR) operations or to feed algae, phytoplankton, or bacteria that enable the production of food, nutritional supplements, feed for livestock, methane, and lipids, which can be converted into biofuel and even the algal plastic called polyethylene furandicarboxylate (PEF) [6]. Thus, the development of the appropriate CO_2_ separation technologies is significant. 

The conventional methods widely used in industrial plants are pressure and temperature swing absorption/adsorption [1,9,10]. However, most of them are very energy-consuming; therefore, their use is limited for environmental and economic reasons. The promising alternatives are membrane technologies, characterized by numerous advantages such as high efficiency, simple design, low energy requirement, easiness of scale-up, and economic and environmental kindliness [9,11,12]. 

The most important factors that must be considered for gas separation membranes are their improved permeability and selectivity, good mechanical strength, and chemical and thermal stability [1,9,13,14]. Both inorganic and organic membranes are promising for CO_2_ separation. However, both types of membranes are characterized by advantages and disadvantages. The perfect solution to overcome their disadvantages would be to combine their decent parameters in the form of hybrid inorganic–organic membranes (limiting the problems associated with the trade-off between permeability and selectivity and high costs of membrane production) [15,16]. This type of membrane could be obtained by incorporating inorganic materials into the polymer matrix [17,18]. The fundamental problem in producing hybrid membranes is the appropriate selection of both the polymer matrix and inorganic additives. Therefore, the authors focused on the promising polymer matrix, PPO, and Fe@MWCNTs as an inorganic additive.

Polyphenylene oxide (PPO) is widely used in numerous branches of the electrical, electronic, and automotive industries due to its high glass transition temperature, which is associated with a high melt processing temperature. The solution to this problem was to propose numerous blends with various polymers, such as polystyrene (Noryl), polyamide (Noryl GTX), polypropylene (Noryl PPX), and a thermoplastic elastomer (Noryl WC). This solution allowed materials with high heat softening temperature, creep resistance, stability in boiling water, electrical resistance, and low mold shrinkage [19]. Poly (2,6-dimethyl-1,4-phenylene oxide) (PPO) is also a polymer with one of the highest gas permeabilities among aromatic polymers (chain packing and densification are suppressed (relatively large fraction free volume (FFV)) by the presence of ether linkage and the absence of polar groups). Therefore, it could be a perfect membrane material because of its excellent mechanical properties, plasticity, and high glass transition temperature. 

Unfortunately, the PPO membranes have moderate selectivity due to the hindered free rotation of the phenyl ring. Because it is a hydrophobic polymer, it is not easily soluble in conventional aprotic solvents. Many electrophilic substitutions, such as bromination, carboxylation, sulfonylation, acylation, etc., were used to improve PPO properties and gas separation characteristics [19,20]. One of the methods of improving the permselectivity of the polymer is the introduction of polar groups, causing stronger interactions between the polymer chains, as in the research on sulfonated polystyrene, Nafion, poly (ether ether ketone), and polyethersulfone [21,22,23]. 

The sulfonation of PPO results in a linear increase in density with ion exchange capacity (IEC) of polymer and also improves the CO_2_/N_2_ permselectivity but unfortunately significantly decreases the CO_2_ permeability [24]. An increase in the IEC of the polymer results in only a slight improvement in the SPPO position relative to the upper Robeson upper bound line for the polymers. 

Therefore, the next solution should be introduced in the form of inorganic–organic hybrid membranes, also called mixed matrix membranes (MMMs). These composites could be obtained by the incorporation of inorganic materials, such as, for instance, silica, metal oxides, CNTs, graphene, nanofibers, zeolites, CMS, MOFs, POFs, etc. [1,25,26,27,28,29,30,31,32,33,34], into the polymer matrix, which may enhance their gas permeability. The introduction of inorganic fillers into the polymer matrix provides new composite materials with enhanced gas transport, mechanical, thermal, electric, or magnetic properties, adjusted by control of the composition, content, and morphology of the filler addition, application of different processing techniques, or by applied modification of both phases [15,16,29,32]. In addition, these composites are easy to produce and characterized by the lower cost of applied materials. However, their efficiency depends mainly on the choice of polymer matrix and inorganic filler and the interaction between these two materials, their compatibility, and good dispersion of the inorganic phase [18]. 

To increase the compatibility between the inorganic additive and the polymer matrix and reduce the size of the introduced particles, multi-wall nanotubes Fe@MWCNTs were proposed as a new carbon filler. Due to their unique optical, mechanical, thermal, magnetic, and electrical properties, CNTs have found many potential applications in various fields, such as catalysis, electronics, military technology, energy, material engineering, or nanomedicine [35,36]. The currently interesting group is CNTs obtained by the synergy of hybrid material components, i.e., carbon nanostructures and magnetic nanoparticles (CEMNPs and MNPs) [37,38]. Many carbon-based fillers such as carbon black (CB), graphene, fullerenes have been used as fillers in hybrid membranes used to separate gas mixtures. Because CNTs, in comparison with other carbon allotropes, have excellent mechanical properties, have a smooth surface and a large specific surface, they will be suitable fillers of inorganic–organic hybrid membranes (better mechanical resistance, numerous selective gas adsorption sites, and highly efficient gas transport) [35,39,40]. 

However, MWCNTs tend to aggregate in a polymer matrix (via π−π interactions and van der Waals forces), which causes a reduction in gas permeability and selectivity of the membrane. This may be because nanotubes randomly dispersed in a polymer matrix will cause transport through discontinuous and tortuous paths of interfacial void spaces between MWCNT aggregates and polymer [35,41,42]. To maximize the selectivity and permeability of hybrid membranes, the MWCNT should be homogeneously dispersed in the polymer matrix and properly positioned concerning the membrane surface. For this purpose, several methods were used, such as functionalization/modification of the external walls of the CNT (covalent and non-covalent functioning) and polymer matrix and the introduction of an electric field [40,41,42,43]. Scientists are currently looking for new methods of increasing the strength and functionality of polymeric materials by creating appropriate composites, which, however, involves solving numerous problems. In recent years, we have been researching new magnetic composites and their potential application in the separation of air components. 

The present publication is a continuation of previous research [44,45,46,47,48,49,50,51], which confirmed the positive effect of the magnetic addition on the mechanical, magnetic, and gas transport properties of the tested composites.

Because Fe@MWCNTs tend to aggregate in the polymer matrix, which may reduce the gas transport properties of the membranes, the use of magnetic field and functionalization of both the inorganic additive and the polymer matrix have been proposed in the framework of this paper. 

In this paper, we report the CO_2_/N_2_ separation results using new, magnetically aligned Fe@MWCNTs/PPO and Fe@MWCNT-OH/SPPO membranes with different filler contents. The higher content of iron characterized the applied Fe@MWCNTs. Aligning of Fe@MWCNTs in hybrid membranes at various magnetic-field inductions and chemical modification of the inorganic filler particles, polymer matrix, and their influence on CO_2_ separation performance was investigated. The obtained membranes were also characterized by a static mechanical performance, *transmission electron microscopy* (TEM), scanning electron microscopy (SEM), thermogravimetry (TGA), *Fourier-transform infrared spectroscopy* (FT-IR), X-ray diffraction (XRD), and vibrating sample magnetometry (VSM). The experimental results were compared with theoretically predicted data based on a three-phase system.

## 2. Materials and Methods

### 2.1. Materials

Poly (2,6-dimethyl-1,4-phenylene oxide) (PPO) was purchased from Sabic Innovative Plastics. *N*-methylpyrrolidone (NMP), chloroform, toluene, chlorosulfonic acid, ferrocene, methanol, iron (II) sulfate heptahydrate, trichloroethylene (TCE), and hydrogen peroxide of 99% purity were supplied by Chemiatrade (Poland). Carbon dioxide (5.5), nitrogen (5.0), and argon (5.0) compressed cylinders were purchased from Air Liquide (Poland). 

### 2.2. Synthesis of Fe@MWCNTs 

Fe@MWCNTs with increased iron content were synthesized in-house via catalytic chemical vapor deposition (c-CVD) protocol using the argon atmosphere as the growth environment and saturated solution of ferrocene (9.6 wt%; catalyst precursor) in toluene as the feedstock. Under the optimized conditions for a given geometry of the horizontal c-CVD reactor (760 °C, argon 99.99% flow rate = 1.8 L/min, the injection rate of the feedstock = 2.8 mL/h), an average diameter of the semi-molten catalyst particles determines the nanotube diameter, whereas the duration of the synthesis controls the nanotube length. Here, iron-saturated Fe@MWCNTs were received as thick films of vertically aligned nanotubes of outer diameter (OD) of 54 ± 31 nm and length (l) of 100 ± 20 µm, a BET surface area (a) of 34 m^2^/g^–1^ and total iron content of 5.80 wt% [36,52]. 

### 2.3. Functionalization of Fe@MWCNTs

Fe@MWCNTs were hydroxylated according to the recipe described in another publication [53]. MWCNTs were added to a solution of iron (II) sulfate heptahydrate in deionized water (DIW). After 15 min of ultrasonication, the hydrogen peroxide aqueous solution (30 wt%) was added in one portion, and then the obtained mixture was ultrasonicated for 12 h. The obtained Fe@MWCNT-OH were separated on the PTFE membrane and then washed with deionized water (DIW) and methanol. After that product was dried three days in a laboratory oven at 80 °C, yielding pure Fe@MWCNT-OH. It was found that their degree of functionalization (df, mmol g^−1^) was 3.5 mmol of the OH group, per 1 g of the nanotube support [53]. At the same time, the Fe: C ratio in the obtained Fe@MWCNT-OH remained unchanged. This indicated that the mechanism of hydroxylation was based mainly on the attack of in situ generated hydroxyl radicals on the centers of crystallographic disorder in the graphene walls.

### 2.4. Sulfonation of PPO

**Figure 1:**
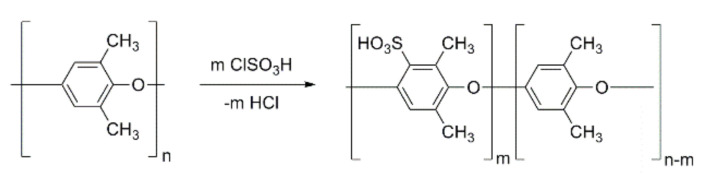
Scheme of PPO sulfonation.

PPO was sulfonated according to the procedure described elsewhere [24,54]. The appropriate amount of PPO was dissolved in chloroform and then sulfonated by dropwise addition of chlorosulfonic acid in chloroform over 1 h with vigorous mechanical stirring at room temperature. The procedure was continued for another hour under a continuous nitrogen purge. Finally, the precipitated polymer was filtered off, washed a few times with deionized water until neutral pH, and then dried (24 h at room temperature and then at 60 °C in a vacuum oven for a further 48 h). The degree of sulfonation of SPPO (20.1%) was determined by the titration method. The scheme of the PPO sulfonation reaction is presented in Figure 1.

### 2.5. Hybrid Membrane Preparation and Characterization 

**Figure 2:**
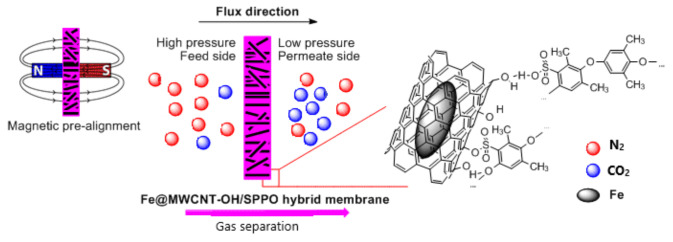
Scheme of composite Fe@MWCNT-OH/SPPO membrane preparation and gas permeation process.

The homogeneous and inorganic–organic hybrid membranes based on a PPO and SPPO matrix and various additions of Fe@MWCNTs or Fe@MWCNT-OH as fillers (CNTs: 0.5–10.0 wt%) were examined. The Fe@MWCNT/PPO membranes were produced by casting ultrasonicated (3 h) Fe@MWCNT dispersions in 2.5% PPO solution in TCE without or with the magnetic field (B = 40 mT or B = 100 mT) of a magnetic coil or two ferrite magnets. In turn, the Fe@MWCNT-OH/SPPO membranes were obtained from the dispersion of Fe@MWCNT-OH in 4% SPPO solution in NMP, obtained by sonication for three hours. The suspensions thus prepared were poured into leveled Petri dishes and evaporated without or with the magnetic field (B = 40 mT or B = 100 mT) of a magnetic coil or two ferrite magnets (Figure 2). By this procedure, a series of membranes with an inorganic content ranging from 0.5–10.0 wt% and thickness 40–60 µm were prepared. Gas (CO_2_ and N_2_) permeability measurements were carried out for membranes using the low-pressure gas permeation analyzer IDP-2 at the temperature of 25 °C [49,50,51]. The analyzed membrane, in the form of a disc (with an effective surface of 19.625 cm^2^), was introduced into the permeation chamber, and then it was rinsed with an appropriate gas. Carbon dioxide and nitrogen were supplied to the system from compressed gas cylinders. In the next step, the appropriate pressure difference (0.3–0.9 MPa) was established and controlled between the high- and low-pressure parts of the chamber using the electronic pressure meter and controller EL-press P-506C. Then, the volumetric flow rate was measured using a Flow-Bus flowmeter (with a range 0–3 mL/min) with a computer data acquisition. These flow-rate data were used to obtain mass transport coefficients (***D***, ***P***, ***S***, and ***α***). 

The mechanical properties of the composite materials were tested on the static testing machine Zwick/Roell Z050. While their magnetic properties were tested using a Lake Shore 7010 vibrating sample magnetometer (VSM).

Cross-sections of the Fe@MWCNT/PPO membranes (cut on a microtome) were characterized using a Transmission Electron Microscope Jeol 1200 at 120 kV acceleration. Earlier Fe@MWCNT examination using TEM revealed that the iron is encapsulated in CNT [52,55]. TGA curves were recorded using a Linseis STA PT1600 thermobalance (Selb, Germany) in a heating rate of 10 °C/min under an argon atmosphere (60 mL/min). Scanning electron micrographs of obtained composite materials were obtained with the SIX HITACHI S-3400N SEM. The samples were analyzed at 5 kV. The prepared membranes were also characterized with FT-IR (Nicolet 6700 (Thermo Electron Corporation, Waltham, MA, USA) with ATR (Attenuated Total Reflection)) and XRD (Rigaku Mini- Flex II diffractometer with Cu Kα radiation).

### 2.6. The Evaluation of Gas Transport Parameters

During the gas permeation experiments, the gas flow rate ***Q_STP_*** was measured and then recalculated into diffusive mass flux in a stationary state ***J_S_***:

(1) $$J_{s}=\frac{Q_{STP}}{A}$$

where: ***Q_STP_*** is a flow rate at the standard condition, $\left[\frac{cm_{STP}^{3}}{s}\right]$, and ***A*** is an active membrane area (cm^2^).

Using ***J_S_***, the permeation coefficient ***P*** was determined according to Formula (2) [44,45]:

(2) $$P=\frac{J_{S\;}l}{\Delta p}$$

where ***P*** is the permeation coefficient [Barrer] $Barrer=\frac{cm_{STP}^{3}\times cm}{cm^{2}\times s\times cmHg}\times 10^{-10}$, ***l*** is a membrane thickness (cm), **Δ*p*** is a gas pressure difference at both sides of the membrane (cmHg), and ***J_s_*** is a diffusive mass flux in a stationary state $\left[\frac{cm_{STP}^{3}}{cm^{2}\times s}\right]$.

The following critical gas transport parameter was the average diffusion coefficient $\bar{D}$ obtained from the stationary state of permeation according to the equation:

(3) $$\bar{D}=\frac{J_{s}\cdot l}{\Delta c}$$

where: ***J_s_*** is a diffusive mass flux in a stationary state ($\left[\frac{cm_{STP}^{3}}{cm^{2}\times s}\right]$), ***l*** is a thickness of the membrane (cm), and **Δ*c*** is a difference in concentrations. After integration of ***J_S_*** with respect to time was received a downstream absorption permeation ***Q^a^ (l, t)*** (total flow of penetrant) from which the concentration difference **Δ*c*** could be determined from the intersection of the asymptote with the ***Q^a^ (l, t)*** axis [44,45]. In turn, the solubility coefficient ***S*** is a measure of the size of sorption in membranes, and it was calculated from Equation (4):

(4) $$S=\frac{P}{\bar{D}}$$

where: ***S*** is a solubility coefficient $\left[\frac{cm_{STP}^{3}}{cm^{3}\cdot cmHg}\right]$.

The last parameter, the ideal selectivity coefficient $\alpha_{CO2/N2}$, could be calculated from the ratio of the obtained permeation coefficients:

(5) $$\alpha_{CO_{2}/N_{2}}=\frac{P_{CO_{2}}}{P_{N_{2}}}$$

where: ***P*_*CO*_2__** and ***P*_*N*_2__** are the permeation coefficients of pure carbon dioxide and nitrogen, respectively.

### 2.7. Modeling of Fe@MWCNT/PPO and Fe@MWCNT-OH/SPPO

In recent years, there has been a growing interest in developing theoretical models designed to predict gas permeation through mixed matrix membranes, which essentially leads to a reduction in the number of experiments and allows studying the influence of various parameters on the final membrane’s performance. However, most of the existing models were applied to predict gas permeability through polymer membranes containing inorganic additives in the form of spherical and platelet particles. Relatively few models considered the presence of tubular particles [56]. The first such model used to predict gas permeation through membranes with tubular particles was the Hamilton–Crosser (HC) model, which is based on the assumption that there is an analogy between thermal conduction and gas permeation in polymer composites [57]:

(6) $$\frac{P_{eff}}{P_{m}}=\frac{5P_{m}+P_{f}+5\varphi \left(P_{f}-P_{m}\right)}{5P_{m}+P_{f}-\varphi \left(P_{f}-P_{m}\right)}$$

where ***P_eff_*** is the effective permeability coefficient of the MMM, and ***P_m_*** and ***P_f_*** are the permeability coefficients of the polymer matrix (continuous phase) and the inorganic filler (dispersed phase), respectively, and ***φ*** is the volume fraction of inorganic filler particles. The next model designed to predict gas permeability through MMMs with tubular filler particles was based on the parallel-series resistance model and named the Kang–Jones–Nair model (KJN) [58]:

(7) $$\frac{P_{eff}}{P_{m}}={\left[\left(1-\frac{cos\theta }{cos\theta +\frac{1}{\alpha }sin\theta }\varphi \right)+\frac{P_{m}}{P_{f}}\left(\frac{1}{cos\theta +\frac{1}{\alpha \;}\;sin\theta }\right)\varphi \right]}^{-1}$$

where ***α*** is the aspect ratio of tubular fillers **(**α=L/d, where ***L*** is the length and ***d*** is the diameter of the nanotube), and ***θ*** is the orientation angle of nanotubes concerning the membrane transport direction.

As the most applied models designed to predict the performance of MMMs, the HC and KJN models assume defect-free interphase between the polymer and filler, resulting from the perfect contact of the two phases. However, due to the different surface chemistry of the polymer matrix and the dispersed inorganic phases, the formation of interface defects, such as voids, in MMM is inevitable. Therefore, a mixed matrix membrane must be considered a three-phase system consisting of a polymer matrix, filler particles, and interfacial voids. Therefore, the HC and KJN models are only suitable for predicting gas transport through membranes, two-phase systems with ideal morphology. In the case of modeling gas permeation through real systems with non-ideal morphology, models based on three-phase systems, such as the one proposed by Chehrazi et al., should be used [56]. The considered model assumes that there is an analogy between mass and heat transfer in polymer composites.

Within this model, two characteristic parameters were introduced, such as “interfacial thickness” (***a_int_***) and “interfacial permeation resistance” (***R_int_***), used to characterize the polymer–nanotube interface. Therefore, the effective gas permeation through a non-ideal nanotube−MMM can be formulated as follows [56]:

(8) PeffPm=3+φ2(daint−1)daint+1+PNT/Pm1+2aintLPNTPm+Laint−13−φ2(d/aint−1)d/aint+1

***P_eff_****, **P_m_***, and ***P_NT_*** are the gas permeability coefficients of the MMM, polymer matrix, and nanotube, respectively. ***φ*** represents the volume fraction of the nanotubes. ***a_int_*** is the thickness of the interfacial region that chemically or mechanically connects the nanotubes and the polymer matrix phases and plays a crucial role in the entire properties of composites; ***L*** and ***d*** are the length and diameter of the nanotube, respectively. In addition, ***P_NT_*** = ***L/R_NT_*** is the gas permeability coefficient of the nanotube. The average absolute relative error (***AARE***) was calculated to compare the predicted results with the experimental data derived from our own research. The average absolute relative error was calculated as follows [50,59]:

(9) $$\%AARE=\frac{100}{NDP}\overset{NDP}{\underset{i=1}{\sum }}\left|\frac{P_{i}^{pred}-P_{i}^{exp}}{P_{i}^{exp}}\right|$$

where ***NDP*** is the number of data points, $P_{i}^{pred}$ is the predicted permeability value, and $P_{i}^{exp}$ is the experimental permeability value. In this paper, the experimental results were compared with the results predicted using the appropriate theoretical model created by Chehrazi et al. [56], enabling the description of gas transport in three-phase systems concerning possible defects. To determine the predicted values ***P_eff_/P_m_***, the relationship given in Equation (8) was used, calculating the permeability of CO_2_, assuming that ***L*** = 10,000 nm, ***d*** = 10 nm, and changing one of the parameters, respectively, while the others remain constant. The values of ***P_NT_/P_m_***, ***φ***, and ***a_int_*** were varied in the ranges 1–10,000, 0.005–0.1, and 50–500 nm, respectively. The values of the changed parameters were selected, considering the smallest possible average absolute relative error %***AARE***. The comparison of the predicted and experimental simulation results is given in Section 3.4.

## 3. Results and Discussion

### 3.1. Magnetic Parameters of Hybrid Membranes

**Figure 3:**
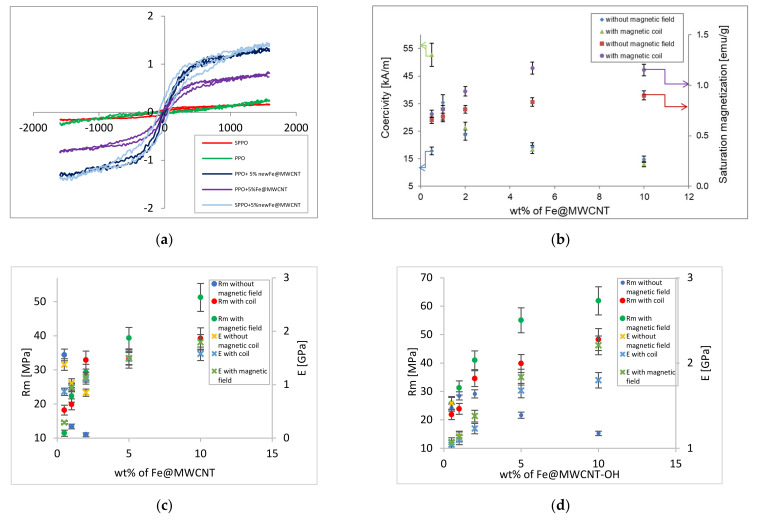
Magnetic and mechanical properties of hybrid membranes: (**a**) hysteresis loops of PPO, SPPO, and Fe@MWCNT/PPO membranes with 5 wt% of two types of Fe@MWCNTs; (**b**) dependence of coercivity and saturation magnetization of hybrid membranes cast in a weaker and stronger magnetic field on Fe@MWCNT loading; (**c**) ***E*** and ***R_m_*** vs. Fe@MWCNT loadings for PPO membranes cast in the absence and in the presence of coil and a stronger magnetic field; (**d**) ***R_m_*** and ***E*** versus Fe@MWCNT–OH loadings for SPPO membranes cast in the absence and in the presence of coil and a stronger magnetic field.

The magnetic properties of hybrid membranes were investigated due to the significant influence of the magnetic field on the creation process of hybrid membranes and their subsequent properties. Magnetic parameters of the hybrid membranes, such as saturation magnetization and coercivity, were determined from the hysteresis loops and are presented in Table 1. Figure 3a,b shows typical hysteresis loops of the membranes without and with two types of fillers and the dependency of magnetic properties on the Fe@MWCNT content. 

**Figure 4:**
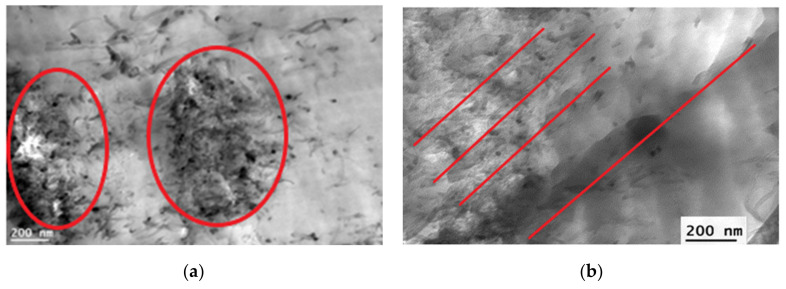
Transmission electron microscopy (TEM) image of cross-sections of Fe@MWCNT/PPO membranes cast in (**a**) a weaker magnetic field and (**b**) a stronger magnetic field. The areas occupied by CNT aggregates and the orientation of CNTs after applying the magnetic field are marked in red.

It was found that the hysteresis loop’s shapes and saturation magnetization point to a PPO and SPPO membrane’s paramagnetic character and slightly ferromagnetic character of membrane containing Fe@MWCNTs, especially for Fe@MWCNT/PPO and Fe@MWCNT-OH/SPPO membranes with higher Fe content (5.80 wt%). For the membranes cast in the absence of a magnetic field, the Fe@MWCNTs were randomly distributed. Whereas, after applying a magnetic field, the CNTs formed chains aligned to the magnetic field lines, especially in a stronger magnetic field. Additionally, the magnetic parameters, such as saturation magnetization and coercivity, changed with the CNT loading. Namely, in a weaker magnetic field, saturation magnetization increased while the coercivity decreased. This phenomenon could be related to the formation of agglomerates (presented in Figure 4a). The coercivity slightly increased or remained relatively unchanged in the stronger magnetic field, which could be caused by more homogeneous dispersion of CNT in a polymer matrix (Fe@MWCNTs are aligned according to the magnetic-field lines—Figure 4b). 

**Figure 5:**
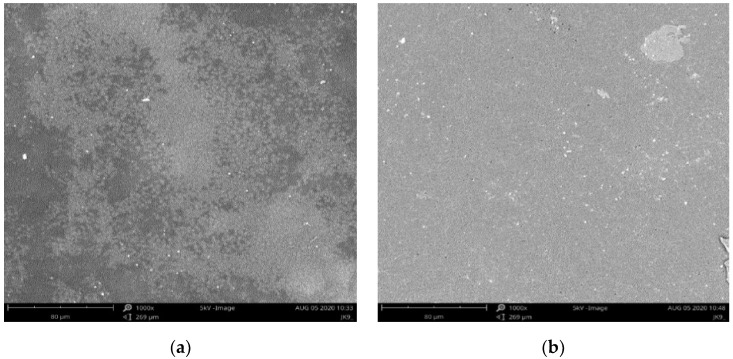

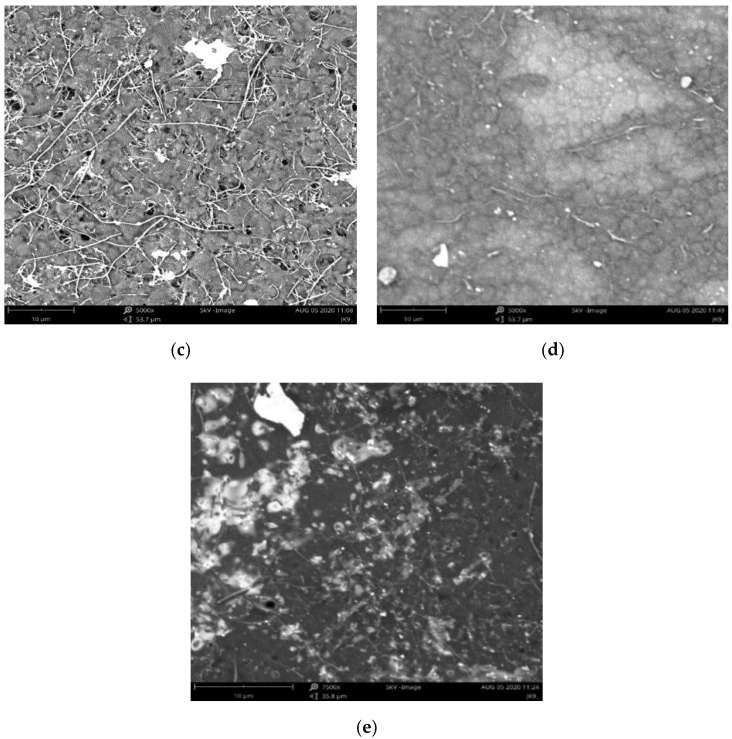
SEM images of: (**a**) Fe@MWCNT/PPO membranes (1 wt%) cast in a weaker magnetic field; (**b**) Fe@MWCNT-OH/SPPO membranes (1 wt%) cast in a weaker magnetic field; (**c**) Fe@MWCNT/PPO (2 wt%) membranes cast in a stronger magnetic field; (**d**) Fe@MWCNT-OH/SPPO (2 wt%) membranes cast in a stronger magnetic field; (**e**) Fe@MWCNT-OH/SPPO (5 wt%) membranes cast in a stronger magnetic field.

Earlier conclusions on the structure of hybrid membranes were also confirmed by SEM analysis. In the case of PPO hybrid membranes cast in a weaker magnetic field, we can observe the formation of CNTs aggregates (Figure 5a). As a result of increasing the magnetic field induction, their better dispersion in the polymer matrix was obtained. However, the more significant addition of CNTs increases the defects caused by the lower adhesion of the nanotubes to the organic matrix (Figure 5c). On the other hand, in the case of hybrid membranes based on the modified SPPO matrix and functionalized nanotubes, greater homogeneity of the tested composites was observed, even with increasing the Fe@MWCNT-OH addition (Figure 5b,d,e), which may indicate an increase in the compatibility between the organic and inorganic phases due to bond formation.

### 3.2. Mechanical Properties of Hybrid Membranes

High mechanical performance of hybrid membranes is required for their future application. Mechanical parameters such as Young’s modulus (***E***) and tensile strength ***(R_m_***) were examined for the hybrid Fe@MWCNT/PPO and Fe@MWCNT-OH/SPPO membranes (Figure 3c,d). The tests showed that both mechanical parameters have changed with the Fe@MWCNT or Fe@MWCNT-OH content. It was found that tensile strength and Young’s modulus values decreased with filler loading for Fe@MWCNT/PPO hybrid membranes cast without the magnetic field. At the same time, the values of ***R_m_*** (growth from 18.45–51.25 MPa) and ***E*** (growth from 0.89–1.79 GPa) were rising with the Fe@MWCNT loading for membranes cast in the magnetic field, especially the stronger one (Figure 3c). While the application of modified polymer matrix and functionalized Fe@MWCNTs (Figure 3d) for non-magnetic cast membranes has led to an initial increase (up to 2 wt%) in ***R_m_*** (29.17 MPa) and decrease in ***E*** (from 1.53 to 0.86 GPa) with increasing additive. This may indicate a potential interaction of the SPPO matrix with modified CNTs. Except that the introduction of sulfonic groups has affected the mechanical properties of modified polymer. Namely, the increase in modulus ***E*** of SPPO membranes could be caused by the increasing density of ionic physical crosslinking between polar ionic sites. Even after using a weaker magnetic field, a positive effect on the mechanical properties was noted.

Moreover, the introduction of a stronger magnetic field during the production of membranes caused an increase in both ***R_m_*** (growth from 25.83 to 61.89 MPa) and ***E*** (growth from 1.07 to 2.21 GPa) with an increase in Fe@MWCNT-OH addition, exceeding values for dense polymer membranes. This may indicate better dispersion and proper alignment of CNTs along the magnetic field lines and the formation of bonds between the organic and inorganic phases. Thus, the improvement of hybrid membrane’s mechanical properties is probably caused by the decrease in polymer chains mobility, the increase in hybrid membrane’s density with the addition of filler, and the appropriate dispersion and alignment of the Fe@MWCNT in the membrane structure. The enhancement of mechanical performance should translate directly into better gas separation properties of the hybrid membranes and their potential future applications. 

**Figure 6:**
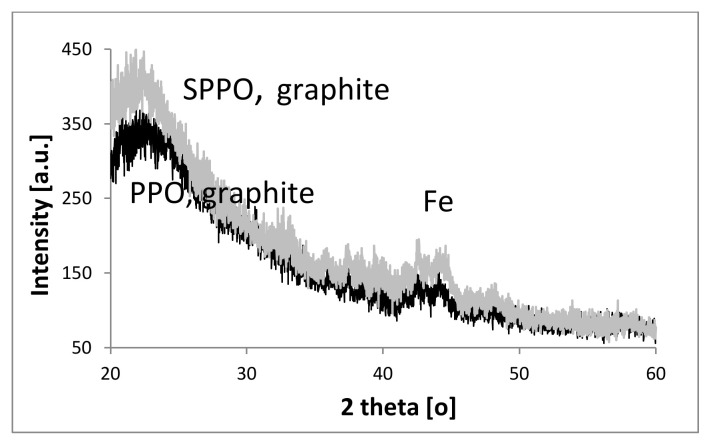
X-ray diffraction spectra for two Fe@MWCNT/PPO and Fe@MWCNT-OH/SPPO hybrid membranes.

The XRD spectra of two types of hybrid Fe@MWCNT/PPO and Fe@MWCNT-OH/SPPO membranes are shown in Figure 6. These spectra consist of characteristic peaks of polymer matrices and inorganic fillers. They are generally similar and indicate a semicrystalline structure of PPO and SPPO matrices (peaks at 21.8 and 22.3°). However, in the case of SPPO, the major peak is slightly shifted to a lower ***2θ*** (a shift of 0.5°). Therefore, the calculated d-spacing for SPPO was slightly larger than that for PPO, and its increase was associated with a rise in peak intensity. All this may indicate a more significant order of macromolecular orientation in the SPPO polymer and a stiffer structure of this membrane. The increase in SPPO peak intensity on the X-ray diffraction spectrum results in an increase in polymer selectivity. It was also found that the presence of peaks corresponding to graphitic shells (25.8°, 42.3°, and 53.7°) and several Fe-phases (42.9° for α-Fe and 44.7° for γ-Fe). However, some peaks could be observed overlapping with peaks characteristic for polymer matrices. Earlier XRD analysis of pure Fe@MWCNTs confirmed the presence of graphitic and Fe-phases [60].

### 3.3. Thermal Properties of Composite Membranes

**Figure 7:**
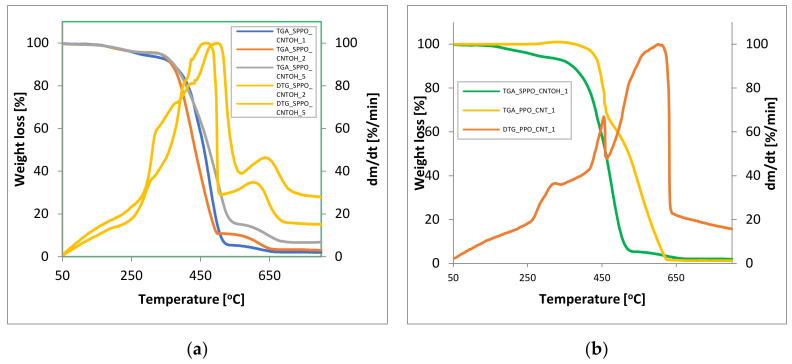
TGA and DTG results for (**a**) Fe@MWCNT-OH/SPPO hybrid membranes with various Fe@MWCNT-OH addition and (**b**) Fe@MWCNT/PPO and Fe@MWCNT-OH/SPPO hybrid membranes with 1 wt% CNT loading.

The thermal stability of PPO and SPPO composite membranes was examined using thermogravimetric analysis, the results of which are presented in Figure 7. In the case of PPO, which is a thermally stable polymer, the degradation of the main chain starts at 465 °C with a maximum of 490 °C. While SPPO shows three-step weight loss: a first one in the range 25–100 °C, which is assigned to the elimination of H_2_O bonded to the sulfonic groups, the second step at about 230 °C corresponds to a loss of sulfonic groups, and the last one at 460 °C is related to the splitting of the main chain [61]. In the case of composite materials, this could be seen as a positive change in thermal stability. Namely, for the Fe@MWCNT/PPO composites could be seen some steps of weight loss: the first one at about 330 °C corresponds to degradation of OH groups, the next one at 465 °C is related to the degradation of PPO polymer main chain, and the last one at 610 °C could be assigned to oxidation of Fe@MWCNTs. While for the Fe@MWCNT-OH/SPPO composites, the following stages of weight loss have been noted: the first one at about 230–270 °C corresponds with a loss of sulfonic groups from the SPPO matrix. The second one in the range 304–330 °C corresponds to the degradation of OH groups from Fe@MWCNT-OH. The next stage at 468–498 °C is related to the degradation of the main polymer chain. The last one at 601–635 °C could be assigned to oxidation of Fe@MWCNT-OH.

It should be noted that with the increase in Fe@MWCNT-OH addition, the maximum of the peaks responsible for the subsequent transformations is shifted towards higher temperatures. This may indicate better thermo-oxidative properties of the obtained hybrids, despite the use of the SPPO matrix. The temperature corresponding to the 5 wt% weight loss during thermo-oxidative degradation increased with increasing content of CNTs in the materials (e.g., from 429 °C for hybrid Fe@MWCNT/PPO with 1 wt% to 437 °C for hybrid PPO with 2 wt% of CNT and from 271 °C for hybrid Fe@MWCNT-OH/SPPO with 1 wt% to 333 °C for hybrid PPO with 5 wt% of CNT-OH). Thus, we can see that TGA results confirmed the thermo-oxidative stability of the analyzed membranes. The thermo-oxidative properties of the obtained composite membranes proved their potential use in CO_2_ separation.

### 3.4. Gas Transport Parameters of Hybrid Membranes 

The crafted composite materials have been tested for potential use in CO_2_ separation from gas mixtures, e.g., with N_2_. The main gas transport parameters for Fe@MWCNT/PPO and Fe@MWCNT-OH/SPPO hybrid membranes were collected in Table 2. Three identical hybrid membranes were measured six times, and the Hartley ***F_max_*** test was performed to analyze the method’s reproducibility. It was found that the obtained standard deviation’s values do not differ from each other in a statistically significant manner (***F_max_*** < ***F_max_* *_o_*** (2.81 < 10.76)), and that is why an average value could be calculated. Procedure reproducibility value was ***CV*** = 8.74% for each series.

Table 3 presents the experimental and theoretical predicted data for the PPO/Fe@MWCNT and SPPO/Fe@MWCNT-OH hybrid membranes prepared under various conditions. The experimental data were compared with the data estimated based on the model by Chehrazi. It was found that the thickness of the interphase ***a_int_*** decreases for membranes based on the SPPO matrix but also in the case of hybrid membranes cast in a magnetic field with increasing magnetic induction (for hybrid membranes based on PPO: ***a_int_*** = 525 nm, SPPO without the magnetic field: ***a_int_*** = 200 nm, SPPO with magnetic field B = 40 mT: ***a_int_*** = 150 nm and SPPO with magnetic field B= 100 mT: ***a_int_*** = 115 nm). This indicates better compatibility and interfacial interaction. The decrease in interfacial thickness ***a_int_*** positively influences the transport properties of the tested hybrid membranes. It was also noted that the value of the ratio of the CO_2_ permeability coefficient through nanotubes to the permeability through the polymer matrix ***P_NT_/P_m_*** increases with the increase in the magnetic field induction (for hybrid membranes based on SPPO without the magnetic field: ***P_NT_/P_m_*** *=* 50, SPPO in the field B = 40 mT: ***P_NT_/P_m_*** = 1000 and SPPO in field B = 100 mT: ***P_NT_/P_m_*** = 10,000). In addition, the value of this ***P_NT_/P_m_*** ratio is twice as high for membranes based on SPPO, compared to PPO (for hybrid membranes based on PPO: ***P_NT_/P_m_*** = 5000 and SPPO: ***P_NT_/P_m_*** = 10,000 in the field B = 100 mT). This is mainly due to the difference in the permeability coefficients of the SPPO and PPO matrix (PPO ***P*_*CO*_2__**: 49.80 and SPPO ***P*_*CO*_2__**: 22.49). It should also be noted that the matching of the experimental and theoretical results for the membranes based on the SPPO matrix is burdened with a more minor error, which indicates more excellent compatibility of the organic and inorganic phases after the modification and much smaller defects of the obtained composites. The used Chehrazi model proved to be suitable for describing the CO_2_ transport through the analyzed membranes. The %AARE error was only 3–7%. Such a good correlation can be associated with considering the possible defects in gas permeability in a non-ideal three-phase system.

**Figure 8:**
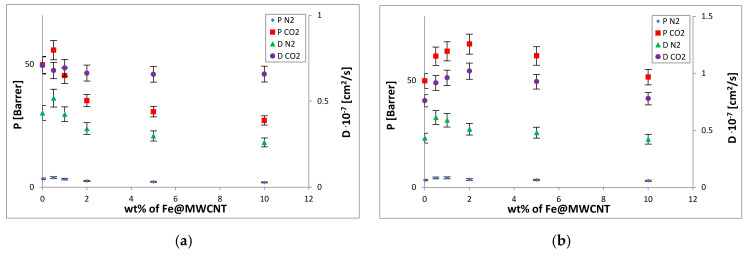

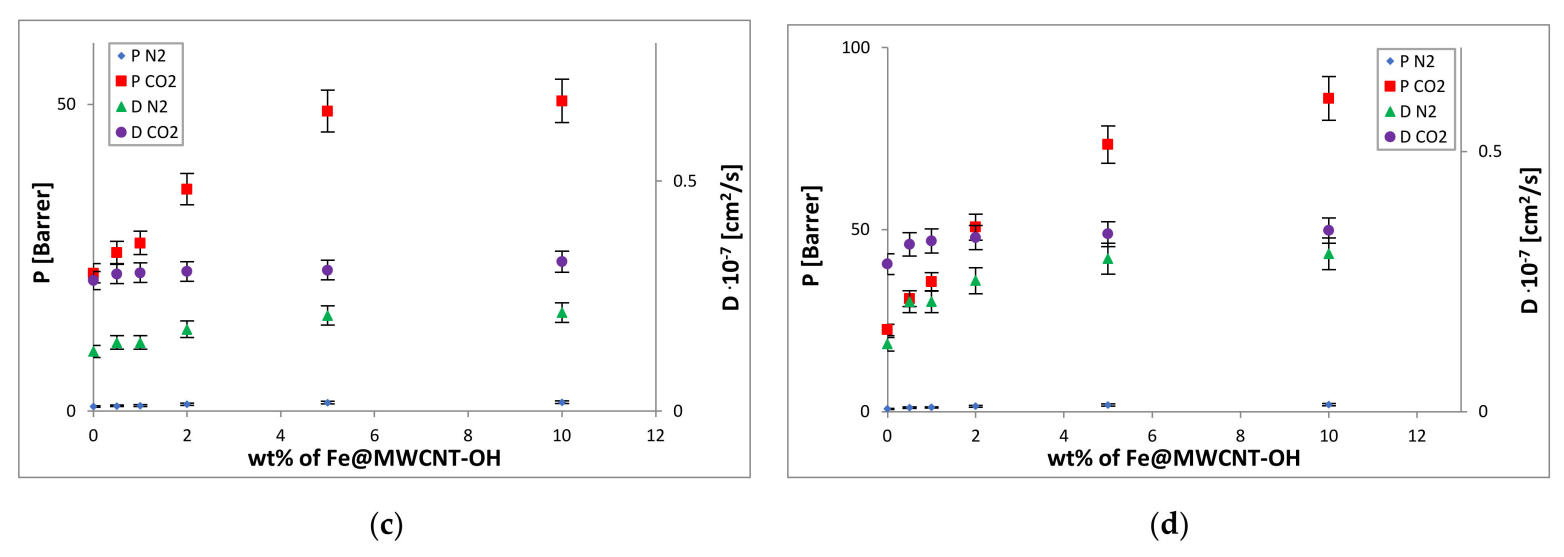
Dependence of the permeation and diffusion coefficients versus: (**a**) Fe@MWCNT loading in the PPO hybrid membranes cast in the absence of the magnetic field; (**b**) Fe@MWCNT loading in the PPO hybrid membranes cast in the presence of the magnetic field; (**c**) Fe@MWCNT-OH loading in the SPPO hybrid membranes cast in the absence of the magnetic field; (**d**) Fe@MWCNT-OH loading in the SPPO hybrid membranes cast in the presence of the magnetic field.

The incorporation of Fe@MWCNT or Fe@MWCNT-OH into the polymer matrices has significantly changed their gas transport properties. It was found that the diffusion and permeation coefficients of the Fe@MWCNT/PPO membranes, cast without a magnetic field, decreased with the increase in CNT loading (Figure 8a). The observed phenomenon could result from CNT clustering that could lead to the creation of a higher content of gas-impermeable crystalline areas than in the pure PPO matrix and cause a reduction in the polymer–filler interface. All those phenomena could decrease free volume and gas molecules’ mobility in the hybrid membrane. It could be seen that a difference in the nitrogen sorption coefficients determined for pure PPO and the Fe@MWCNT/PPO membrane was not significant. Hence, the reduction in gas permeability (compared with pure PPO) could be mainly connected with the decrease in a diffusion coefficient, which is consistent with the clustering mentioned above and the effect of increased crystallinity. To improve the gas transport properties of hybrid membranes, enhancing the Fe@MWCNTs dispersion in a polymer matrix was also necessary. For this purpose, the magnetic casting of hybrid membranes was applied to enable Fe@MWCNTs vertical alignment, especially in a stronger magnetic field (Figure 8b).

**Figure 9:**
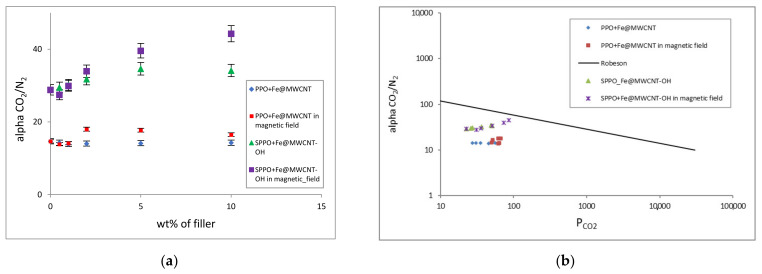
Dependence of a selectivity coefficient **α_*CO*_2_/*N*_2__** versus: (**a**) filler concentration in the various hybrid membranes and (**b**) permeation coefficient ***P*_*CO*_2__** regarding the Robeson upper bound line.

It turned out that the membranes prepared in this way had higher carbon dioxide permeabilities and improved selectivities (Figure 9). It could be then seen that magnetic casting can be the appropriate method to improve the efficiency of hybrid membranes for CO_2_/N_2_ separation. The CO_2_ diffusion, permeation, and sorption coefficients increased until the 2.0 wt% loading (***D*_*CO*_2__** from 7.60 to 10.19, ***P*_*CO*_2__** from 49.80 to 66.99, and ***S*_*CO*_2__** from 6.55 to 6.70). Unfortunately, the decrease in coefficients (***D*_*CO*_2__** from 10.19 to 7.78, ***P*_*CO*_2__** from 66.99 to 51.57, and ***S*_*CO*_2__** from 6.70 to 6.64) was observed for higher filler additions. However, their values were more significant than coefficients of polymer dense PPO membrane (***D*_*CO*_2__** 7.60, ***P*_*CO*_2__** 49.80, and ***S*_*CO*_2__** from 6.55). In turn, the magnetic field application only slightly improved the selectivity coefficient **α_*CO*_2_/*N*_2__** (from 14.67 to 17.99). Therefore, for further research, it would be necessary to modify both the polymer matrix and CNTs, such as PPO sulfonation and CNT functionalization by OH functional groups, to enhance their compatibility (via excessive hydrogen bonding and possible sulfonate (ester) moieties) and affinity to CO_2_. 

**Figure 10:**
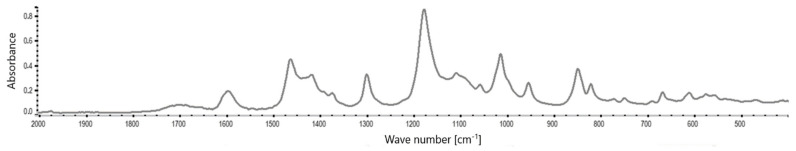
FT-IR spectrum of SPPO polymer membrane.

FT-IR analysis was used to confirm the process of sulfonation of the PPO membrane matrix. The results of the FT-IR analysis are shown in Figure 10. It was observed that the bands’ intensity at 775 cm^−1^ and 825 cm^−1^ decreases or disappears. This relates to the substitution of the H atom in the aromatic ring by a sulfonic group. It could also be observed new bands at 665 cm^−1^ and 1060 cm^−1^, which are assigned to the C-S stretching vibration and S = O stretching vibration of the SO_3_^-^ group, respectively. At the same time, the -SO_3_- band at 1180 cm^−1^ overlaps with the aromatic ether band. It was found that the sulfonation of PPO caused the increase in separation coefficients **α_*CO*_2_/*N*_2__** (from 14.87 to 28.83) and decrease in CO_2_ (***P*_*CO*_2__** from 49.80 to 22.49) and N_2_ (***P*_*N*_2__**: from 3.40 to 0.78) permeabilities. This phenomenon could be caused by the increase in density and decrease in mobility of the polymer chains (stronger inter-chain interactions). It was noted that the modification of the organic and inorganic phases alone made it possible to obtain composites with improved gas transport properties, but the additional introduction of the magnetic field significantly improved their properties. The introduction of functionalized CNTs and the presence of a stronger magnetic field led to the creation of membranes characterized by the increased permeation, diffusion, sorption, and even selectivity coefficients **α_*CO*_2_/*N*_2__** (from 28.83 to 44.28) with the Fe@MWCNT-OH loading rise (Table 2, Figure 8 and Figure 9). The permeability coefficient ***P*_*CO*_2__** has significantly increased with the nanotube content for Fe@MWCNT-OH/SPPO membranes (***P*_*CO*_2__** from 22.49 to 86.02). This behavior could be caused by the increase in polymer chain’s stiffness, the increase in the interface between the polymer and carbon additive, and eventually the larger free volumes and higher mobility of gas molecules. While the permeability coefficient ***P*_*N*_2__** slightly increased with the nanotube content for Fe@MWCNT-OH/ (from 0.78 to 1.94). That could be associated with a slight increase in the diffusion coefficient ***D*_*N*_2__** values (from 0.13 to 0.30). It was also noted that the nitrogen sorption coefficient ***S*_*N*_2__** values were smaller and their changes with the CNT loading were within the error range. At the same time, ***D*_*CO*_2__** and ***S*_*CO*_2__** values were higher and increased with inorganic additive loading (***D*_*CO*_2__**: from 2.84 to 3.48 and ***S*_*CO*_2__**: from 7.92 to 24.70). This increase in CO_2_ permeability through the functionalized membranes may result from an increase in both coefficients, but especially of the sorption coefficient. This is probably due to a lower crystallinity and the increase in free volume. 

Moreover, symmetrical carbon dioxide molecule has a constant electric quadrupole moment. Such a quadrupole can interact with the three-dimensional structure of the ordered “suprastructures” formed by Fe@MWCNTs in the polymer matrix of hybrid membranes, which causes CO_2_ to enter its cavities. On the other hand, nitrogen, having a much smaller quadrupole moment, does not interact with the structure mentioned above. The structure characteristics and the presence of oxygen (hydroxyl) groups may affect the adsorption efficiency and separation of gases such as CO_2_/N_2_ in these structures. Reducing the size, introducing Fe atoms, but most of all oxidation of the CNTs surface and sulfonation of polymer matrix causes a significant increase in CO_2_/N_2_ separation efficiency. This is due to the electrical nature of the CO_2_ molecule, which has a quadrupole moment and therefore interacts much more strongly with the polar groups than does nitrogen molecules. In addition, the use of MWCNTs with a more developed structure allows for a significant improvement in the adsorption properties, which may be caused by an increase in the interaction energy of the CO_2_ molecules with the “suprastructures” formed by Fe@MWCNTs in the polymer matrix. It was observed (Figure 9b) that with the increasing filler addition, Fe@MWCNT/PPO and Fe@MWCNT-OH/SPPO composite membranes were characterized by increasing CO_2_ permeability and selectivity. It was also stated that the measurement points approached Robeson’s upper bound line (Figure 9b) along with the increase in filler content. Thus, it was found that more productive membranes were obtained by modifying both the inorganic CNT additive and the organic polymer matrix.

## 4. Conclusions 

Hybrid inorganic–organic membranes were successfully synthesized from PPO, SPPO, and Fe@MWCNTs or Fe@MWCNT-OH as fillers. It was found that the incorporation of nanofillers with increased iron content (5.80 wt%) into the polymer matrix had significantly improved gas transport (D, P, S, and **α_*CO*_2_/*N*_2__**), magnetic, thermal, and mechanical parameters of analyzed membranes, especially after application of magnetic casting and chemical modification of inorganic and organic phase. The magnetic field application in the production of membranes enabled the CNT’s alignment and improved dispersion and positively affected their gas transport properties. However, only its combination with the functionalization of both phases (enhanced interphase compatibility) allowed the selectivity coefficient **α_*CO*_2_/*N*_2__** to increase to 44.28 and the permeation coefficient ***P*_*CO*_2__** to 86.02. The mechanical (***E*** and ***R_m_***) parameters of the tested membranes were improved by the increase in the nanofiller addition (Fe@MWCNTs or Fe@MWCNT-OH) and selection of the appropriate type of polymer matrix (an increase in ***R_m_***: from 25.83 to 61.89 MPa and ***E***: from 1.07 to 2.21 GPa for Fe@MWCNT-OH/SPPO). It was also stated that the thermo-oxidative stability of investigated composites increased with the increasing content of CNTs. The model developed by Chehrazi et al. proved to be suitable for describing the CO_2_ transport through analyzed hybrid membranes (the %AARE error was only 3–7%.). These membranes seem appropriate for CO_2_ separation from gas mixtures, especially after introducing chemical modifications and external magnetic field (increase in inter-phase compatibility and the affinity to CO_2_). This type of solution in the form of selective membranes for CO_2_ separation, e.g., from flue gases from coal combustion, may find future applications in the power industry.

## Figures and Tables

**Table 1:** Magnetic parameters of Fe@MWCNT/PPO hybrid membranes with various CNT loading prepared at various magnetic field inductions.

wt% Fe@MWCNT	B (mT)	Coercivity (kA/m)	Saturation Magnetization (emu/g)
0.5	0	17.821	0.654
1	0	35.436	0.690
2	0	23.616	0.761
5	0	19.224	0.834
10	0	14.772	0.899
0.5	40	52.704	0.716
1	40	30.912	0.763
2	40	26.212	0.939
5	40	18.336	1.170
10	40	13.015	1.152
0.5	100	18.240	0.790
1	100	18.624	0.886
2	100	21.888	1.256
5	100	23.616	1.343
10	100	30.005	1.681

**Table 2:** CO_2_ and N_2_ gas transport properties of (a) Fe@MWCNT/PPO membranes prepared in the absence and (b) in the presence of the stronger magnetic field, (c) Fe@MWCNT-OH/SPPO membranes prepared in the absence, and (d) in the presence of the stronger magnetic field.

**(a)**								**(b)**					
**Membrane Fe@MWCNT/ PPO** **with Fe@MWCNTs** **(wt%)**	***α*** _***CO*_2_/*N*_2_**_	**N_2_**			**CO_2_**			**N_2_**			**CO_2_**		
		$\bar{D}$	***P***	***S***	$\bar{D}$	***P***	***S***	$\bar{D}$	***P***	***S***	$\bar{D}$	***P***	***S***
		**10^8^ (cm^2^/s)**	**(Barrer)**	**10^2^ (cm^3^_STP_/** **cm^3^cmHg)**	**10^8^ (cm^2^/s)**	**(Barrer)**	**10^2^ (cm^3^_STP_/** **cm^3^cmHg)**	**10^8^ (cm^2^/s)**	**(Barrer)**	**10^2^ (cm^3^_STP_/** **cm^3^cmHg)**	**10^8^ (cm^2^/s)**	**(Barrer)**	**10^2^ (cm^3^_STP_/** **cm^3^cmHg)**
0.0	14.67	0.43 ± 0.04	3.40 ± 0.31	7.87 ± 0.71	7.60 ± 0.46	49.80 ± 2.99	6.55 ± 0.39	0.43 ± 0.06	3.40 ± 0.31	7.87 ± 0.71	7.60 ± 0.46	49.80 ± 2.99	6.55 ± 0.39
0.5	14.28	0.52 ± 0.05	3.90 ± 0.35	7.54 ± 0.68	8.32 ± 0.50	55.78 ± 3.34	6.70 ± 0.40	0.61 ± 0.06	4.42 ± 0.40	7.21 ± 0.68	9.15 ± 0.55	61.35 ± 3.68	6.70 ± 0.40
1.0	13.89	0.42 ± 0.05	3.27 ± 0.29	7.71 ± 0.69	7.39 ± 0.44	45.42 ± 2.73	6.15 ± 0.37	0.59 ± 0.05	4.50 ± 0.40	7.64 ± 0.69	9.60 ± 0.57	63.58 ± 3.81	6.62 ± 0.39
2.0	14.03	0.34 ± 0.03	2.51 ± 0.23	7.37 ± 0.66	5.66 ± 0.34	35.26 ± 2.11	6.23 ± 0.37	0.51 ± 0.05	3.72 ± 0.33	7.31 ± 0.66	10.19 ± 0.61	66.99 ± 4.02	6.57 ± 0.39
5.0	14.14	0.30 ± 0.03	2.17 ± 0.19	7.30 ± 0.66	4.88 ± 0.29	30.73 ± 1.84	6.30 ± 0.38	0.48 ± 0.05	3.47 ± 0.31	7.23 ± 0.66	9.27 ± 0.55	61.45 ± 3.68	6.63 ± 0.39
10.0	14.28	0.26 ± 0.03	1.90 ± 0.17	7.31 ± 0.66	4.08 ± 0.24	27.14 ± 1.63	6.65 ± 0.40	0.42 ± 0.04	3.13 ± 0.30	7.40 ± 0.66	7.78 ± 0.46	51.57 ± 3.09	6.64 ± 0.40
**(c)**								**(d)**					
**Membrane Fe@MWCNT-OH/ SPPO** **with Fe@MWCNT-OH ( wt%)**	***α*** _***CO*_2_/*N*_2_**_	**N_2_**			**CO_2_**			**N_2_**			**CO_2_**		
		$\bar{D}$	***P***	**S**	$\bar{D}$	***P***	***S***	$\bar{D}$	***P***	***S***	$\bar{D}$	***P***	***S***
		**10^8^ (cm^2^/s)**	**(Barrer)**	**10^2^ (cm^3^_STP_/** **cm^3^cmHg)**	**10^8^ (cm^2^/s)**	**(Barrer)**	**10^2^ (cm^3^_STP_/** **cm^3^cmHg)**	**10^8^ (cm^2^/s)**	**(Barrer)**	**10^2^ (cm^3^_STP_/** **cm^3^cmHg)**	**10^8^ (cm^2^/s)**	**(Barrer)**	**10^2^ (cm^3^_STP_/** **cm^3^cmHg)**
0.0	28.83	0.13 ± 0.01	0.78 ± 0.07	6.00 ± 0.54	2.84 ± 0.18	22.49 ± 1.57	7.92 ± 0.55	0.13 ± 0.01	0.78 ± 0.07	6.00 ± 0.54	2.84 ± 0.18	22.49 ± 1.57	7.92 ± 0.55
0.5	29.47	0.15 ± 0.01	0.88 ± 0.08	5.87 ± 0.53	2.98 ± 0.21	25.86 ± 1.81	8.67 ± 0.61	0.21 ± 0.02	1.13 ± 0.10	5.35 ± 0.45	3.22 ± 0.21	31.04 ± 2.17	9.65 ± 0.67
1.0	30.19	0.15 ± 0.01	0.91 ± 0.08	6.08 ± 0.55	3.01 ± 0.22	27.44 ± 1.92	9.11 ± 0.64	0.21 ± 0.02	1.19 ± 0.10	5.63 ± 0.46	3.28 ± 0.22	35.67 ± 2.49	10.87 ± 0.76
2.0	31.80	0.18 ± 0.02	1.14 ± 0.10	6.39 ± 0.58	3.04 ± 0.24	36.21 ± 2.53	11.92 ± 0.83	0.25 ± 0.02	1.49 ± 0.13	5.93 ± 0.48	3.35 ± 0.24	50.69 ± 3.54	15.14 ± 1.05
5.0	34.60	0.21 ± 0.02	1.41 ± 0.12	6.80 ± 0.61	3.07 ± 0.25	48.92 ± 3.42	15.95 ± 1.12	0.29 ± 0.03	1.85 ± 0.16	6.30 ± 0.54	3.41 ± 0.25	73.37 ± 5.13	21.49 ± 1.50
10.0	34.14	0.21 ± 0.02	1.48 ± 0.13	6.91 ± 0.62	3.25 ± 0.26	50.60 ± 3.54	15.57 ± 1.09	0.30 ± 0.03	1.94 ± 0.17	6.40 ± 0.55	3.48 ± 0.26	86.02 ± 6.02	24.70 ± 1.73

**Table 3:** Experimental and theoretical predicted data for the PPO/Fe@MWCNT and SPPO/Fe@MWCNT-OH hybrid membranes, prepared under various conditions.

Membrane	*φ*	Experimental Data	Theoretical Data	Parameters for Simulation	*AARE* (%)
		*P_eff_/P_mCO_*_2_	*P_eff_/P_mCO_*_2_		
PPO/Fe@MWCNT in a strong magnetic field	0.005	1.232	1.039	***a_i__nt_*** = 525 nm; ***P_NT_/P_m_*** = 5000	7.02
	0.010	1.277	1.079		
	0.020	1.345	1.156		
	0.050	1.234	1.383		
	0.100	1.035	1.742		
SPPO/Fe@MWCNT-OH without magnetic field	0.005	1.150	1.103	***a_int_*** = 200 nm; ***P_NT_/P_m_*** = 50	3.10
	0.010	1.220	1.206		
	0.020	1.610	1.409		
	0.050	2.175	2.004		
	0.100	2.250	2.951		
SPPO/Fe@MWCNT-OH in a weak magnetic field	0.005	1.323	1.157	***a_int_*** = 150 nm; ***P_NT_/P_m_*** = 1000	3.13
	0.010	1.403	1.313		
	0.020	1.852	1.622		
	0.050	2.501	2.529		
	0.100	2.925	3.974		
SPPO/Fe@MWCNT-OH in a strong magnetic field	0.005	1.380	1.209	***a_int_*** = 115 nm; ***P_NT_/P_m_*** = 10,000	3.71
	0.010	1.586	1.417		
	0.020	2.254	1.830		
	0.050	3.263	3.041		
	0.100	3.825	4.974		

## Data Availability

The data presented in this study are available on request from the corresponding author. The data are not publicly available due to the extremely large size.
